# 24. An analysis of the National Institutes of Health All of Us Research Database: Sociodemographic Disparities Among Patients Who Received Vaccinations

**DOI:** 10.1093/ofid/ofab466.226

**Published:** 2021-12-04

**Authors:** Ding Quan Ng, Stanley Jia, Christine Cadiz, Cheryl Wisseh, Megan H Nguyen, Joyce Lee, Sarah McBane, Lee Nguyen, Alexandre Chan, Keri Hurley-Kim

**Affiliations:** University of California Irvine, Irvine, California

## Abstract

**Background:**

The National Institutes of Health All of Us (AoU) research program is building a diversified database of 1 million+ adult subjects. With this database, we seek to describe the sociodemographic characteristics of those with documented vaccinations.

**Methods:**

The AoU recruited subjects ≥ 18 years beginning in 2018. Eligible subjects were subsequently divided into five vaccine cohorts based on their vaccine history [influenza, hepatitis B (HepB), pneumococcal (Pneu) < 65, Pneu ≥ 65, human papillomavirus (HPV)]. The vaccine cohorts were compared to the general AoU cohort. Subjects in the influenza cohort had documented influenza vaccinations from 09/2017-05/2018. Other vaccine cohorts comprised subjects with ≥ 1 lifetime record(s) of vaccination by 12/2018. The Pneu < 65 and ≥ 65 cohorts comprised those who received pneumococcal vaccination before or after (inclusive) 65 years old, respectively. Descriptive statistics for all cohorts were generated using survey and electronic health record (EHR) data.

**Results:**

We analyzed 315297 subjects in the AoU dataset R2020Q4R2. The cohort sizes were: influenza (n=15346), HepB (n=6323), HPV (n=2125), and Pneu (< 65 n=15217; ≥65 n=15100). For all vaccine cohorts, comparing the 95% confidence intervals (CIs), the proportions of whites and non-Hispanics/Latinos were statistically higher than the general AoU cohort, the largest being from the Pneu ≥ 65 cohort (Table 1). For educational attainment, the Pneu < 65 (36.5%) had the smallest proportion of college or advanced degree graduates while the largest was observed in the Pneu ≥ 65 cohort (59.0%). The proportions of subjects with < &10k in annual household income (AHI) were largest among Pneu < 65 (17.1%) and smallest among Pneu ≥ 65 (3.8%). In contrast, the largest proportion of subjects with ≥ &100k AHI was among Pneu ≥ 65 (25.3%) and the smallest among Pneu < 65 (15.8%).

Table 1. Sociodemographic characteristics of subjects in the All of Us research program based on vaccine receipt

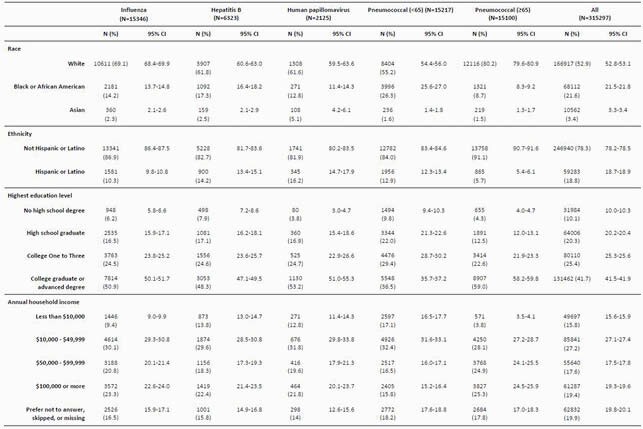

**Conclusion:**

Racial and ethnic disparities in vaccinations were apparent. Pneumococcal vaccination at age 65 years and above was more prevalent among white, non-Hispanic/Latino subjects who were also more educated and affluent. Conversely, those receiving pneumococcal vaccination before age 65 years were less educated and had lower AHI.

**Disclosures:**

**All Authors**: No reported disclosures

